# 

**Published:** 1857-02

**Authors:** 


☞ This number of our Journal will be sent to a number of physicians who are not subscribers, and while we should be pleased to consider them as such—having had numerous assurances that we are publishing a journal that is worthy of patronage—we have no disposition to annoy them by continuing to send it, in opposition to their wishes; they will, therefore, do us the favor to return the number if they are not disposed to be considered subscribers.

---

☞ At the suggestion of a friend, we would again state, that this Journal is intended to be accessible to the communications of any respectable medical man, who is pursuing his profession in a regular way; and contributions are earnestly solicited, with the assurance that they will, at all times, be treated with proper respect and courtesy, without, however, any assumption of responsibility upon the part of the editors, in reference to the views entertained by their correspondents; and with the distinct understanding, that whatever appears in the Journal, will be subject to the courteous criticism of the profession.

---

☞ We can furnish the back numbers of the present volume to new subscribers, if desired; as will be perceived upon examination, it commences in September.

☞ We would desire to call attention to the prospect of additional interest connected with our Journal, from the correspondence and reports of Dr. Hart of New Orleans, (now in Paris, where he will remain some time longer) an allusion to which, has been made in the late correspondence of Professor Westmoreland, who will shortly leave Paris for Edinburgh, from which point, the latter will continue his interesting communications.

---

### RECEIPTS.

J. T. House, N. C., 2d vol.; J. M. Couch, Ga., 1st and 2d vol.; J. P. Taylor, Ga., 1st and 2d vol.; Jos. T. Bond, Ga., 2d vol.; Jno. Coston, Ga., 2d vol.; Wm. Burch, Ga., 1st and 2d vol.; Jno. Stone, Ga., 1st vol.; Wm. A. Bennett, Ala., 2d vol.; Jno. Venable, Ga., 2d vol.; O. H. P. Slaton, Ga., 1st vol.; J. M. McMichael, Ga., 1st vol.

### TO CORRESPONDENTS.

☞ All Communications pertaining to the Editorial Department of the Journal, should be addressed to the Editors.

☞ All Communications, having reference to the Subscription, the Advertising or the Financial Department, should be addressed to the Treasurer, J. G. WESTMORELAND, M. D., Dean of the Faculty of the Atlanta Medical College.

